# Primary Malignancy in a Supernumerary Testicle Presenting as a Large Pelvic Mass

**DOI:** 10.1155/2017/4529853

**Published:** 2017-10-22

**Authors:** Justin Noroozian, Daniel Farishta, Daniel Ballow, Joseph Sonstein, Eduardo Orihuela, Eduardo Eyzaguirre

**Affiliations:** ^1^School of Medicine, University of Texas Medical Branch, Galveston, TX, USA; ^2^Division of Urology, University of Texas Medical Branch, Galveston, TX, USA; ^3^Department of Pathology, University of Texas Medical Branch, Galveston, TX, USA

## Abstract

Supernumerary testis, also known as polyorchidism, is a condition characterized by the presence of more than two testes. Another condition of the testes is seminoma, a common cause of testicular germ cell tumor. A 35-year-old male was transferred to our hospital with a diagnosis of abdominal mass causing abdominal pain. On physical exam, he had a palpable undescended left testicle in the left inguinal canal, which was determined to be seminoma. The mass was surgically removed, and the patient underwent chemotherapy. The report discusses his workup, treatment, and outcome. This case illustrates an unusual presentation of supernumerary testis with the extra testis harboring a seminoma. When presented with a case of testicular cancer with no tumor noted in the palpable testes, malignancy in an extranumerary testicle should be considered in the differential.

## 1. Case Presentation

**Figure 1:**
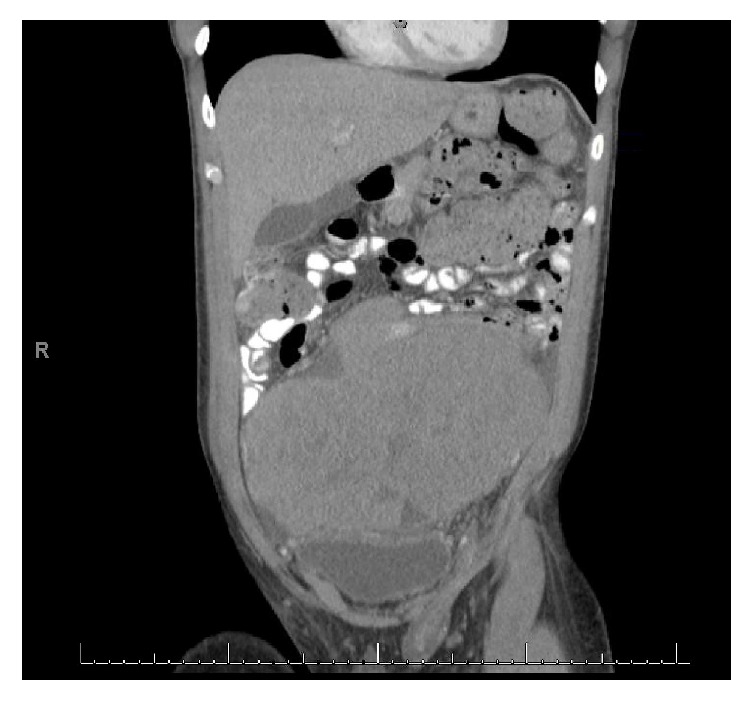
Coronal CT showing mass discreet from undescended left testicle.

**Figure 2:**
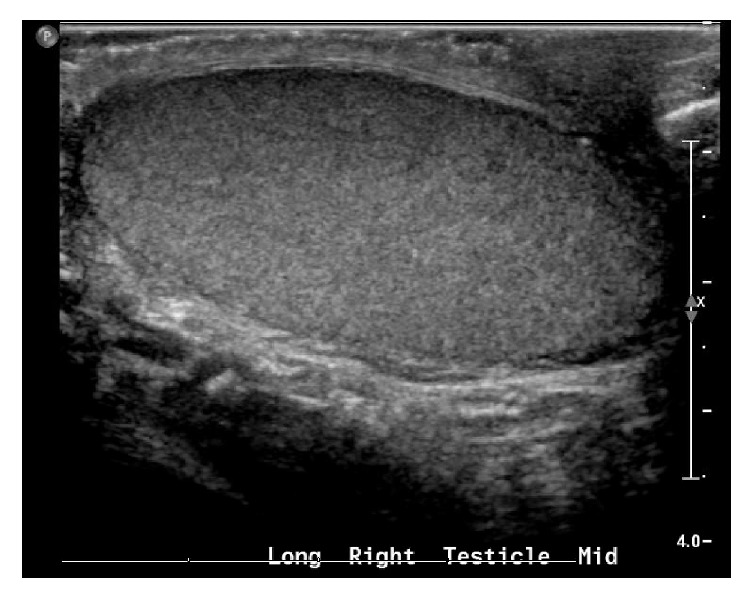
Ultrasound of normal right testicle.

**Figure 3:**
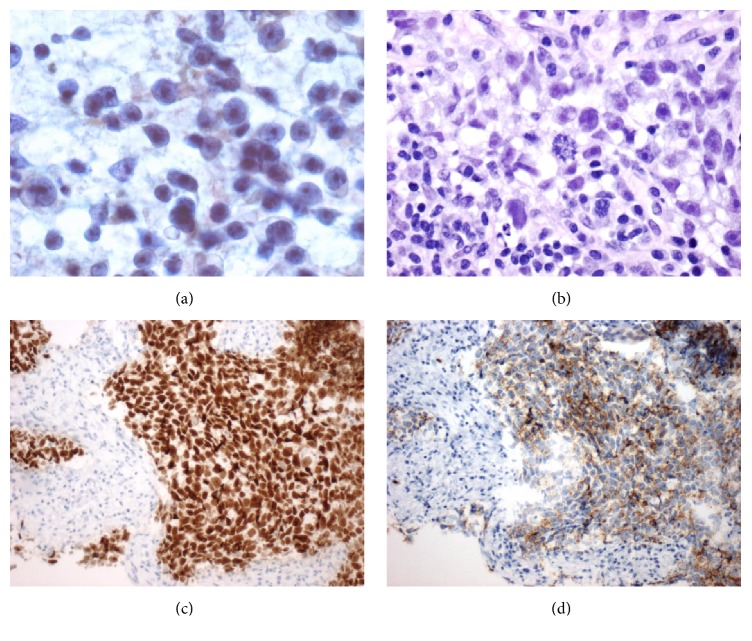
FNA smears and core biopsy of abdominal mass. Malignant cells with high nuclear to cytoplasmic ratio, pleomorphic nuclei, and macronucleoli, singly and in loose clusters, in a background of small lymphocytes (Papanicolaou stain, ×400) (a); core biopsy section shows similar tumor cells (H&E stain, ×400) (b); tumor cells show immunoreactivity to Oct-3/4 (c) and c-kit (d).

**Figure 4:**
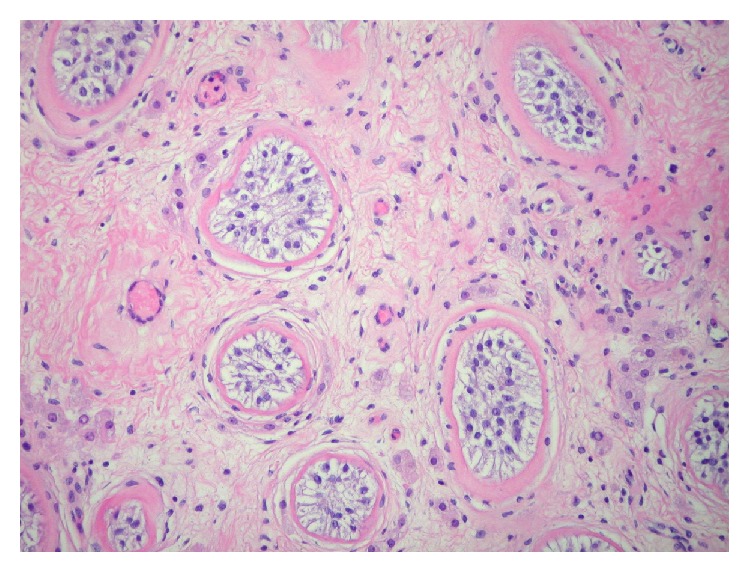
Left testicle, orchiectomy. Atrophic seminiferous tubules with small diameters, absence of spermatogenesis, increased numbers of Sertoli cells, and thickened basement membranes (hematoxylin-eosin stain, ×200).

**Figure 5:**
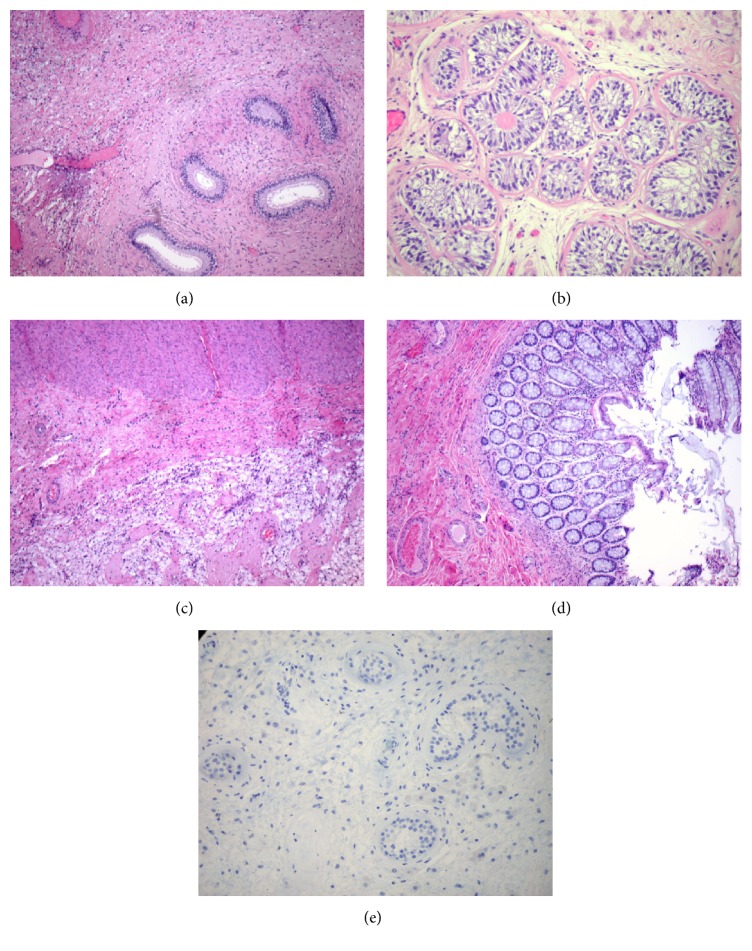
Retroperitoneal mass, excision. Nonviable tumor with extensive hyalinization and xanthogranulomatous reaction, and epididymis (a) (H&E, ×40); testicular parenchyma with extensive tubular atrophy, Sertoli cell hyperplasia, and clusters of small seminiferous tubules with pseudostratified Sertoli cells. Some tubules show Sertoli cell-produced basement membrane-like material inside the hypoplastic tubules (b) (H&E, ×200); colonic wall adjacent to nonviable tumor showing xanthogranulomatous reaction (c); colonic mucosa with no pathologic changes (d) (c and d: H&E, ×40). Immunoperoxidase stain with antibody against OCT 3/4 shows absence of germ cell neoplasia in situ in atrophic tubules (e) (DAB and hematoxylin counterstain, ×200).

**Figure 6:**
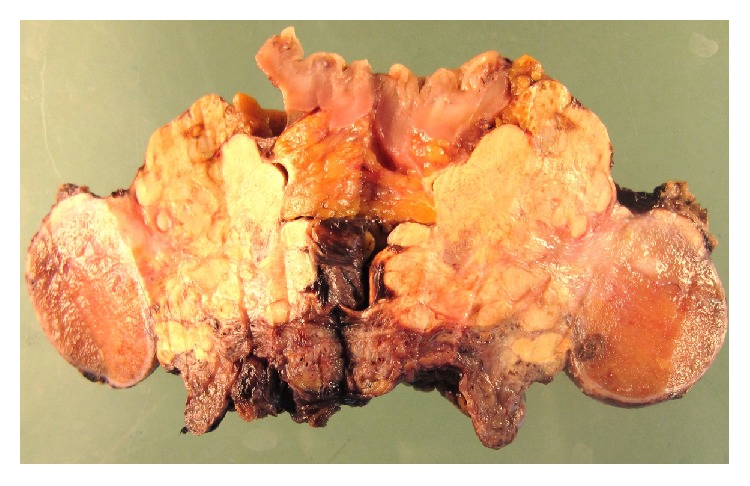
Gross photograph of retroperitoneal mass. A 4.3 cm lobulated solid white-yellowish mass with fibrous bands and extensive necrosis is seen adjacent to the undescended testis and a segment of colon.

Patient is a 35-year-old male who was transferred to our hospital with a diagnosis of abdominal mass after presenting to an outside hospital (OSH) for abdominal pain. Patient reports a three-month history of abnormal bowel movements and a 3-week history of an enlarging, palpable abdominal mass. A CT scan obtained at the OSH revealed a 14 × 18 × 20 cm abdominopelvic mass causing ureteral obstruction and bilateral hydronephrosis, with no retroperitoneal lymphadenopathy (Figure 1). Physical examination revealed the patient had an undescended left testicle, palpable in the left inguinal canal. Serum tumor markers for testicular cancer were obtained and beta HCG was found to be 10 mIU/ml (upper limit of normal), AFP was within normal limits, and LDH was elevated at >6x the upper limit of normal. The patient underwent a percutaneous biopsy of the mass by interventional radiology, and he subsequently underwent cystoscopy with bilateral ureteral stent placement and radical left inguinal orchiectomy. The percutaneous biopsy returned positive for metastatic seminoma (Figure 3). The left radical orchiectomy specimen contained no tumor (Figure 4). Ultrasound of the right testis revealed no lesions (Figure 2). CT of the chest was negative for metastatic disease. The patient underwent 4 cycles of BEP. Two cycles were given without bleomycin, the first due to PFT abnormalities and the last due to rash development with bleomycin during the 3rd cycle. Follow-up imaging revealed decrease in mass size to 5 × 3.4 × 5 cm and an incidental pulmonary embolism (PE), which was asymptomatic. He was treated for the PE with therapeutic low molecular weight heparin for 3 months. PET imaging demonstrated pet avidity in the region of the residual mass, so the patient was taken back to the operating room, 5 months after completion of chemotherapy. Pathology from this specimen showed no viable tumor (Figures 5 and 6) but did contain testicular parenchymal tissue. Based on the totality of pathologic findings, we believe this to be a case of primary pure seminoma arising in a supernumerary testis rather than metastatic spread. The patient tolerated the procedure well and had no complications, pain, or evidence of recurrence at 1-month and 6-month follow-up appointments.

## 2. Discussion

Testicular germ cell tumors are the most prevalent malignancy in males between the ages of 15 and 35 years [1]. Furthermore, seminomas comprise nearly half of all testicular germ cell tumors [2] with an incidence rate of 3.7 and 0.9 cases per 100,000 persons for whites and blacks, respectively [3]. Typical patients that have seminoma present with a noticeable painless testicular lump and abnormal results on semen analysis; patients may also be subfertile [4]. A seminoma is diagnosed when histopathology reveals pure seminoma, without an increase in serum alpha-fetoprotein levels [1]. A sequela of seminoma is metastasis to the retroperitoneal lymph nodes [5]; thus, it is important to begin medical management soon after seminoma is diagnosed. Undescended testis is a known risk factor for testicular germ cell tumors. Less commonly, another congenital abnormality of the male testis, polyorchidism, also known as supernumerary testis, may be associated with germ cell tumor of the testis. This combination of supernumerary testis and seminoma has only been documented approximately 100 times in medical literature [6]. Furthermore, supernumerary testes have more than a 30% incidence of histological abnormalities and 4–7% of polyorchidism cases may be associated with malignancy [7]. We present a patient with both seminoma and polyorchidism, and we believe the tumor arose solely within the supernumerary testis.
